# Physical Activity in Children's Health and Cognition

**DOI:** 10.1155/2018/8542403

**Published:** 2018-06-25

**Authors:** Zan Gao, Senlin Chen, Haichun Sun, Xu Wen, Ping Xiang

**Affiliations:** ^1^School of Kinesiology, The University of Minnesota, Minneapolis, MN, USA; ^2^School of Kinesiology, Louisiana State University, Baton Rouge, LA, USA; ^3^College of Education, University of South Florida, Tampa, FL, USA; ^4^Department of Physical Education, College of Education, Zhejiang University, Hangzhou, Zhejiang, China; ^5^College of Education and Human Development, Texas A&M University, College Station, TX, USA

## 1. Introduction

Physical inactivity is increasingly becoming a major public health concern in many industrialized countries. Afforded by technological advancement and the low demand of physical exertion in living, people in the modern societies, youth and adults alike, have been accustomed to the more sedentary lifestyles. Inadequate physical exertion, in conjunction with the easy access to energy dense diet, has led to dramatic increases in the prevalence of obesity [1]. Obesity is a chronic disease that is associated with morbidities (e.g., cardiovascular disease and type 2 diabetes) and mortality. In the youth population, in particular, cost-effective preventive interventions with reasonable suitability in settings such as schools or preschools are warranted.

Regular participation in physical activity helps reduce the health risk of childhood obesity and the associated chronic diseases. In addition, recent studies suggest that increased participation in physical activity influences cognitive functions in children, including executive functioning (e.g., working memory and cognitive flexibility) and brain health [2–6]. However, these studies mainly targeted older children and adolescents, while more evidence is needed to enlighten the relationships between physical activity, health outcomes, and cognition during the critical period of child development, particularly early childhood [4, 7]. Therefore, this special issue addresses this literature gap in an attempt to stimulate more research efforts in such an important area. It consists of one systematic review and 12 original research articles that studied pediatric population in early childhood as well as healthy children and children with special needs.

## 2. Physical Activity Research in Early Childhood

In this special issue, we specifically examined the effects of physical activity on various health outcomes and cognition in early childhood, as well as the correlates and determinants of physical activity and sedentary behaviors during this critical period of human development. N. Zeng et al. conducted a systematic review to summarize the existing evidence concerning the effects of physical activity programs on preschool children's motor skills and cognitive development. Of the 15 studies, 12 reported statistically significant effects of physical activity on motor skills (e.g., locomotor skills and object control skills) and cognitive development (i.e., language learning, academic achievement, attention, and working memory). No study found adverse effects of the physical activity programs. Therefore, the authors concluded that physical activity was positively associated with both motor skills and cognitive development in preschool children, calling for more research with large representative samples as well as the dose-response evidence in early childhood.

In a randomized controlled trial, S. Xiong et al. examined the effects of a structured physical activity program on preschool children's executive functions and perceived physical competence as compared to traditional recess. The intervention was a 30-minute weekly physical activity program for 3 months. The study suggests that the intervention group had significantly greater increases in executive functions, yet there were no greater increases in perceived physical competence compared to the control group. The authors conclude that it is meaningful and practical to offer structured physical activity programs at day care centers.

G. Zhao et al. features a 15-year longitudinal study protocol to assess the relationship between objectively measured physical activity and cognitive development across the lifespan: childhood, adolescence, and adulthood. Moreover, the study attempted to explore how physical activity affects cognitive development via modification of blood neurotrophins, such as insulin-like growth factor 1 (IGF-1) and brain-derived neurotrophic factor (BDNF). In this protocol, a total of 500 preschool children (3.5–5.5 years) were recruited and followed over 15 years. Six triennial waves of data collection and additional clinical assessments were conducted on variables including (1) anthropometric and physical fitness assessments; (2) 7-day accelerometer-based physical activity; (3) cognition; and (4) plasma IGF-1 and BDNF concentrations. The study may contribute to the literature regarding the effect of a longitudinal physical activity intervention on cognitive development across the lifespan.

H. Fang et al. examined the relationships between objectively measured physical activity and physical fitness in preschoolers (3–5 years). A total of 346 children (201 boys) in Shanghai, China, completed physical fitness assessments (i.e., triceps skinfold thickness, grip strength, tennis throwing, sit and reach test, standing long jump, balance beam, and 10 m/20 m shuttle run), while physical activity on seven consecutive days was captured via ActiGraphGT3X+ accelerometers. The findings suggested that MVPA appears to be an effective and reliable predictor of boys' body composition, muscular strength and power, agility, and aerobic fitness, as well as girls' agility, aerobic fitness, and balance.

Y. Fang et al. investigated the visual motor integration (VMI) development among 151 Chinese preschoolers from 4 to 6 years. Observational evidence revealed that children's VMI skills increased quickly at age 4 and peaked at age 5 and then decreased from age 5 or 6. Children's motor coordination and cognitive flexibility were found to be correlated with VMI development, which in turn was related to visual perception at age 4 and inhibitory control at the beginning of age 5. No statistically significant relationship between children's working memory and VMI was observed. The authors concluded that preschool children's VMI development varied with age. The study provided valuable insights for health professionals with regard to preschool children's VMI development.

P. Sedlak et al. compared the long-term changes of body weight, height, and body mass index (BMI) among healthy Czech Republic children aged 3–6 years (*N* = 3526) across the late 1950s–1960s, 1990s, and 2014–2016. The findings indicated that BMI did not change significantly in boys between ages of 3, 5, and 6 and in girls between 3 and 6 years old during these decades. However, subscapular, suprailiac, triceps, midthigh, and above patella skinfold thicknesses significantly increased in 2014–2016 as compared to 1950s–1960s. Moreover, children in 1990s and 2014–2016 showed similar growth patterns on subcutaneous fat. Of note, higher adiposity was seen in trunk while muscle mass decreased in the lower extremity, suggesting the decline of physical activity among children in the Czech Republic. More physical activity interventions in this population, therefore, are warranted.

## 3. Physical Activity Research in Healthy Children

In this special issue, scholars examined the effects of various physical activity interventions on health outcomes and cognitive functions and investigated the correlates and determinants of health-related physical fitness from childhood to adolescence. In particular, E. W. Evans et al. assessed the feasibility, acceptability, and preliminary effect of wearable monitors on school-age children's physical activity. In 2014, 32 fifth graders from a low-resource middle school were enrolled in Phase I. Waist-worn Fitbit Zip monitors were used for 4 weeks to test the feasibility (i.e., adherence: wear time of ≥8 hrs/day) and acceptability. Phase II occurred in 2015, with 42 sixth graders assigned to (1) Fitbit + goal and incentive-based intervention, (2) Fitbit only, and (3) control condition, to test the feasibility of the Fitbit Charge and its effect on physical activity over six weeks. Findings indicated that average adherence was 64.1% in Phase I, while it was 73.4% and 80.2% for children in Phase II for the Fitbit + intervention and Fitbit only groups, respectively. Notably, there were no statistically significant group differences in MVPA/steps following treatment. The study concluded that the wearable physical activity monitors did not increase school-aged children's physical activity.

P. H. Kulinna et al. examined the effect of an acute physical education dance session on elementary school children's selective attention. Eight Year 5 and Year 6 classrooms were randomly assigned to either intervention or comparison (*n* = 104) groups. Children in the intervention group (*n* = 88) received a dance-based physical education lesson while children in the comparison group (*n* = 104) participated in regular classroom work. Selective attention was assessed via the D2 Test of Attention before and after the interventions. Significant improvements in the intervention group were observed in the total number of items processed and concentration performance, suggesting that an acute bout of aerobic dance delivered during a regular physical education lesson may boost elementary children's cognitive processing speed and concentration performance.

J. Harris et al. investigated the effects of a 4-week coordinated-bilateral physical activity (CBPA) intervention on school-aged children's attention and concentration. A total of 116 fifth graders from two elementary schools were assigned to two different intervention groups or control group. All groups completed pre- and postassessments for D2 Test of Attention. The CBPA group showed significant increases in processing speed, focused attention, concentration performance, and attention span over the course of 4 weeks. Notably, the CBPA group showed significant improvements in concentration performance and attention span as compared to the Fitbit-O. However, no significant differences were observed in attention parameters between the Fitbit-O and the control groups. It was concluded that a 4-week CBPA program was effective in improving children's attention and concentration.

X. Zhu et al. examined grade-level prevalence of physical activity and screen time in association with academic burden among Chinese school-aged children. The sample consisted of 48,118 children from 12 grades, 17 municipal counties/districts, and 534 schools in Shanghai, China. Data on self-reported physical activity, perception of physical activity sufficiency, factors for activity insufficiency, homework hours, and screen time in a typical week were collected. Findings suggested that children had lower physical activity and screen time, as well as higher homework time during 6th, 9th, and 12th grades. In addition, academic burden was seen as the primary reason for physical inactivity in these Chinese students. While those who did not report academic burden were more likely to meet physical activity guidelines, most of whom did not meet recommendations for screen time. More strategies aiming to increase young children's physical activity and reduce screen time in school settings are needed.

J. M. Cosgrove et al. examined the relationships among grit (i.e., passion and perseverance to overcome barriers), school attendance, and academic achievement in 397 adolescents in the southern United States, 77.1% of whom had Hispanic origin. Results showed that adolescents with higher grit scores and better school attendance had better academic performance and achievement.

In addition, L. Yin et al. examined the criterion validity of simple muscle strength test (SMST) indicators while assessing whole body muscle strength in Chinese children. Two hundred and forty children aged 10 to 12 years were enrolled in the study. The SMST indicators, including hand-grip, knee bent push-up, back muscle strength, sit-up, leg muscle strength, and standing long jump, were measured. Results revealed that leg muscle and back muscle strength exhibited the highest validity scores, while sit-ups, hand-grip, and standing long jump demonstrated the lowest. Despite males tending to have the highest validity for leg muscle strength measurement, females showed the highest validity for back muscle strength measurement.

## 4. Physical Activity Research in Children with Disabilities

Several studies also explored the relationships between physical activity and health outcomes and cognition in children with disabilities. In detail, M. Zhao and S. Chen assessed the effects of a 12-week structured physical activity program on social interaction and communication in children with autism spectrum disorder (ASD). Fifty children with ASD were randomly assigned to either experimental (*n* = 25) or control (*n* = 25) groups. Significant improvements in communication, cooperation, social interaction, and self-control subdomains were observed in the experimental group, while no substantial differences were found in the control group after a 12-week intervention. It was concluded that the 12-week physical activity program positively affected social skills, communication, prompt response, and frequency of expression for children with ASD.

L. Waltersson and E. Rodby-Bousquet investigated physical activity level in young adults with cerebral palsy (CP), in relation to their previous physical activity levels, pain, and gross motor function during adolescence. Prospective cohort data from the Swedish National CP Registry (CPUP) that included 129 individuals born in 1991–1993 were extracted at three separate time points: 14–16 years old, 17-18 years old, and 19–22 years old. The findings revealed that physical activity level during early adulthood was positively linked with physical activity during adolescence but not related to pain and gross motor function. Moreover, the physically active individuals with CP during adolescence were more likely to be energetic during early adulthood. Therefore, more strategies aiming to increase physical activity during adolescence are needed among individuals with CP.

Finally, J. Wyszyńska et al. compared physical activity level and screen time between children and adolescents with intellectual disability (ID) and their peers without ID, while also investigating the prevalence of hypertension (HPT) among the ID population. The ID and non-ID groups included 568 participants aged 7 to 18 years, respectively. The findings demonstrated that physical activity level among children with ID were 4 times lower than their non-ID peers. Of note, children with ID were more likely to develop HPT, and screen time seemed to be an important predictor of HPT among children and adolescents. More intervention strategies are warranted to promote physical activity and reduce screen time among this population.

Overall, the 13 studies enclosed in this special issue feature experimental research and descriptive research for design (e.g., correlational, survey, and developmental research). It is noteworthy that all studies reported very positive findings of the health benefits of physical activity in various pediatric populations, in both healthy individuals and those with disabilities. These studies advance the current knowledge in this area. We hope that these articles will be perceived as scholarly engaging and intellectually provocative. We also hope that more studies with innovative approaches and advanced techniques [8, 9] to continue addressing this important research area and promote physically active lifestyles in children and adolescents. We firmly believe that the goal of this special issue is achieved if these hopes become reality.
